# Prevalence and relationship of olfactory dysfunction and tinnitus among middle- and old-aged population in Korea

**DOI:** 10.1371/journal.pone.0206328

**Published:** 2018-10-23

**Authors:** Do-Yang Park, Hyun Jun Kim, Chang-Hoon Kim, Jae Yong Lee, Kyungdo Han, Ji Ho Choi

**Affiliations:** 1 Department of Otolaryngology, Ajou University School of Medicine, Suwon, Republic of Korea; 2 Department of Medicine, Yonsei University Graduate School, Seoul, Republic of Korea; 3 Department of Otorhinolaryngology, Yonsei University College of Medicine, Seoul, Republic of Korea; 4 Department of Otorhinolaryngology-Head and Neck Surgery, Soonchunhyang University College of Medicine, Bucheon Hospital, Bucheon, Republic of Korea; 5 Department of Biostatistics, College of Medicine, The Catholic University of Korea, Seoul, Republic of Korea; University of Sao Paulo Medical School, BRAZIL

## Abstract

Olfactory dysfunction and tinnitus are age-related otorhinolaryngological disorders with a high prevalence in the elderly population and share several common clinical features. However, there is no study investigating the relationship between these two diseases. We studied the prevalence of olfactory dysfunction and tinnitus among Koreans and studied the relationship between these two diseases based on the Korean National Health and Nutrition Examination Survey. The subjects of this study were enrolled from the Fifth Korean National Health and Nutrition Examination Survey (2010–2012, n = 25,534). Data of subjects aged 40 years and older who underwent physical examination and completed a self-reported questionnaire and other anthropometric variables were statistically analyzed. Odds ratios were calculated to identify the relationship between olfactory dysfunction and tinnitus, using multiple logistic regression models. Older males, non-smokers, non/lower alcohol drinker groups exhibited the relationship between olfactory dysfunction and tinnitus. Metabolic syndrome and mental health problems were associated with both olfactory dysfunction and tinnitus. After adjusting for confounding factors, olfactory dysfunction was significantly associated with tinnitus (OR 1.318). There was a dose-response relationship between tinnitus severity and the odds of olfactory dysfunction (ORs for mild, moderate and severe tinnitus were, respectively, 1.134, 1.569 and 2.044). Additional molecular genetics and animal studies are needed to determine the shared pathophysiology of the two diseases.

## Introduction

Olfactory dysfunction is defined as the inability or decreased ability to perceive smell, and is a highly prevalence disease.[1] Olfaction is closely related to quality of life.[2] Disorders of olfaction lead to loss of appetite, cognitive decline due to unpleasant odors or hazardous substances.[3, 4] Furthermore, patients diagnosed with olfactory dysfunction may be at risk of developing nutritional disorders, and be exposed to dangers of food spoilage, leakage of gas, smoke, and pollution. Therefore, olfactory dysfunction is not a life- threatening problem in itself, but can be lethal challenge to patients by increasing the risk of infectious disease and environmental disaster.[2, 5, 6] Furthermore, olfactory dysfunction is thought to be associated with depression, anxiety, and other psychiatric disorders.[7–11] Olfactory dysfunction is thought to arise from upper respiratory infections, chronic sinus and nasal diseases, head trauma, and neurodegenerative diseases.[12] Olfactory dysfunction is generally prevalent in elderly persons compared with younger individuals. Olfactory dysfunction decreases the quality of life related to eating, emotional and social relationships closely related to well-being.[11] Therefore, complications of olfactory dysfunction must be especially well-controlled in prevention, diagnosis, and treatment of middle- and old-aged population. According to the nationwide survey, the prevalence of self-reported olfactory problems was 1.4% in the United States [3] and 4.5% in Korea.[13]

Tinnitus is defined as a perception of sound in the absence of any corresponding external source.[14] The prevalence of tinnitus was 25.3% in the United States [15] and 21.4% in Korea [16] according to a nationwide survey. Similar to olfactory dysfunction, high prevalence in elderly population is closely related to social relationships and well-being.[17, 18] Tinnitus is also affected by psychiatric disorders, such as anxiety, depression, and suicidal ideation. [19–21]

Otorhinolaryngological disorders are frequently closely related with each other. Ear, nose, and throat are located closely and share a common passage. Many infectious diseases are inter-related and affected by each other. For instance, chronic laryngitis and/or eustachian tube dysfunction are affected by posterior nasal drip of rhinosinusitis. Therefore, organ-specific and syndromic disease understanding, and study of organs affecting each other provides a comprehensive insight into otorhinolaryngological problems, for appropriate intervention.

Each disease risk factor in olfactory dysfunction and tinnitus has been investigated in multiple studies. However, there are no studies associating the two diseases. Both olfactory dysfunction and tinnitus appear as two unrelated multifactorial disorders in the elderly population, without a clearly defined etiology and pathophysiology. The main causes of olfactory dysfunction include post-viral upper respiratory infection, nasal/sinus disease, and head trauma, with unclear pathophysiology. Moreover, the olfactory dysfunction is closely related to mental health issues. Tinnitus also has a multifactorial pathophysiology associated with infection and trauma similar to olfactory dysfunction, and psychiatric problems.[19, 20, 22–25] Olfactory dysfunction and tinnitus share common features such as high prevalence in old age, and the effect of physiological and psychiatric factors. These two diseases may interact with each other. However, there are no studies about the relationship between these diseases. Therefore, we investigated the prevalence of olfactory dysfunction and tinnitus, and analyzed the relationship between olfactory dysfunction and tinnitus.

## Materials and methods

### Study populations

Korea Centers for Disease Control and Prevention, in conjunction with the Korean Society of Otorhinolaryngology-Head and Neck Surgery and other societies, have periodically evaluated the medical history and clinical data of the Korean population in the Korean National Health and Nutrition Examination Survey (KNHANES).

The KNHANES was developed as nationwide survey that has been conducted by the Korea Centers for Disease Control and Prevention investigated the health and nutritional status of general Korean population since 1998. KNHANES uses a multistage cross-sectional, stratified sampling method without overlapping subjects. Four medical experts, including an otolaryngologist, visited and conducted the clinical examinations nationwide with specially equipped mobile examination vehicle. A single visit was required for each participant to the examination vehicle. All the questionnaires, examinations and samplings were performed at the single visit. The Korean Society of Otorhinolaryngology-Head and Neck Surgery educated the residents of the survey team for standardization of the examination. Our study was performed using data from the 2010 to 2012 data set. (n = 25,534) Among 25,534 individuals, 23,621 (92.51%) agreed to participate in otorhinolaryngologic questionnaire and examination and included subjects aged more than 40 years (n = 12,618). The mean age of the 12,618 subjects was 59.26±11.94 years (range, 40–97 years) and the ratio of male to female was 1:1.32.

### Ethical considerations

The survey protocol was approved by the institutional review board of the Korea Centers for Disease Control and Prevention (IRB No. 2010-02CON-21-C, 2011-02CON-06-C, and 201201EXP-01-2C). The participants provided written informed consent at baseline.

### Assessment of olfactory dysfunction and tinnitus

The olfactory questionnaire asked whether the participants have had problems with their sense of smell during the past three months (Table 1). Participants provided positive and negative responses suggesting hyposmic and normosmic status, respectively.

**Table 1:** Survey questionnaire and prevalence of olfactory dysfunction and tinnitus.

	%^a^
**Have you had problems with the sense of smell during the past three months?**	
**Yes**	**6.4±0.3**
**No**	93.6±0.3
**Have you heard any ringing, buzzing, roaring, or hissing sounds without an external acoustic source in the past year?**	
**Yes**	**23.3±0.6**
**No**	76.7±0.6
**Do these sounds bother you?**	
** No**	14.6±0.5
** A little annoying**	7.8±0.3
** Very annoying**	0.9±0.1

^a^ Estimated rate, adjusted with weight values

Participants inquired about their tinnitus symptoms within the past year. Examiners were instructed to record ‘yes’ if a participant reported hearing an unusual noise at any time in the past year. Participants who responded positively were then asked about the resulting annoyance in their lives. The participants were considered to have tinnitus if the severity was ‘annoying’ or ‘severely annoying’ (Table 1).

### Assessment of demographic characteristics and lifestyle habits

Medical history and lifestyle habits were recorded based on self-reported questionnaires. Patients were categorized according to smoking history as current smokers, ex-smokers, or nonsmokers. Participants who consumed more than 30 g alcohol/day were considered heavy drinkers. Regular exercise was defined as strenuous physical activity performed for at least 20 min at a time at least three times per week. Participants who had life partners were designated as spouses and job status was defined by employment. Residency was categorized urban or rural according to the official address of participants. Education was classified as high when the participant graduated high school. Low income was categorized corresponding to the lowest quartile of annual household income.

### Assessment of anthropometric and laboratory measurements

Weight, height, and waist circumference (WC) were measured by a well-trained medical survey team. Body-mass index (BMI) was calculated as weight (kg)/height (m^2^). Obesity was defined as a BMI ≥25 kg/m^2^, as recommended by the International Obesity Task Force (IOTF) and the World Health Organization (WHO) Regional Office for the Western Pacific Region for Asian individuals.[26] Blood samples, for the determination of serum levels of biochemical markers, were obtained from the antecubital veins of the participants following a 10– to 12-h overnight fast.

### Assessment of mental health status

Physical and mental health status was evaluated for levels of perceived stress (‘‘light or no” or ‘‘some or heavy”), depressed mood for at least 2 weeks (yes, no), suicidal ideation for the last 12 months (yes, no), and self-rated health status (excellent or good, fair, and poor or very poor).

### Definition of metabolic syndrome

Metabolic syndrome was defined according to the criteria proposed by the American Heart Association and the National Heart, Lung, and Blood Institute together with the International Diabetes Federation in 2009.[27] Participants were diagnosed with metabolic syndrome based on at least three of the following criteria: (1) WC more than 90 cm in men and 80 cm in women; (2) fasting blood sugar more than 100 mg/dL or taking medication for elevated blood glucose level; (3) fasting triglyceride more than 150 mg/dL or taking medication for lowering cholesterol; (4) High-density lipoprotein (HDL)-cholesterol less than 40 mg/dL in men and less than 50 mg/dL in women or taking medication for lowering cholesterol; and (5) Systolic blood pressure (SBP) more than 130 mmHg and/or diastolic blood pressures (DBP) more than 85 mmHg or taking an antihypertensive drug for patients with a history of hypertension.

### Statistical analysis

Statistical analyses were performed using the SAS survey procedure (ver. 9.3; SAS Institute, Cary, NC, USA) for the complex sampling design and sampling weights from the KNHANES, as well as to provide nationally representative prevalence estimates. The procedures included unequal probabilities of selection, oversampling and non-response.

The prevalence and 95% confidence intervals (CIs) for tinnitus were calculated. In the univariate analysis, the Rao-Scott chi-square test (using PROC SURVEYFREQ in SAS) and logistic regression analysis (using PROC SURVEYLOGISTIC in SAS) were used to test the association between tinnitus and risk factors. Participants’ characteristics were analyzed using means and standard errors for continuous variables and numbers and percentages for categorical variables. Simple and multiple logistic regression analyses were used to examine the association between tinnitus and olfactory dysfuction.

We adjusted for age and gender (model 1) and then for the variables in model 1 plus smoking status, alcohol intake, regular exercise, income level, and education level (model 2), and finally adjusted model 2 for BMI, metabolic syndrome, diabetes mellitus (DM), hypertension (HTN), and stress level (model 3). The *p* values were two-tailed, and a *p* < 0.05 was considered significant.

## Results

### Prevalence, associated factors of olfactory dysfunction and tinnitus in the study population

Questionnaire-based, self-reported prevalence of olfactory dysfunction and tinnitus was 6.4±0.3% and 23.3±0.6%, respectively. The prevalence of subjects with tinnitus and related discomfort (a little annoying, very annoying) symptoms was 7.8±0.3%, 0.9±0.1%. (Table 1)

Age, BMI, WC, smoking, drinking, exercise, spouse, job, residency, education, income, stress, depressive mood, and suicidal ideation, potentially associated with olfactory dysfunction and tinnitus, were analyzed in the two diseases. Olfactory dysfunction was associated with age, WC, spouse, job, urban residency, education, income, moderate to severe stress, depressive mood and suicidal ideation under the category of lifestyle habits, or anthropometric and laboratory measurements. Tinnitus was associated with age, BMI, spouse, job, education, income, moderate-to-severe stress, depressive mood and suicidal ideation. (Table 2) Participants with older age, no spouse, no job, low education, and low income, severe stress, depressive mood, and suicidal ideation showed a high prevalence of the two diseases.

**Table 2:** Analysis of factors potentially associated with olfactory dysfunction and tinnitus (n = 12,618).

**Parameter**	**Olfactory dysfunction**				**Tinnitus**		
	No (n = 11,753)	Yes (n = 865)	*p*-value	No (n = 9,501)		Yes (n = 3,117)	*p*-value
Age (years)	55.7±0.2	60.7±0.6	<0.0001*	55.1±0.2		59.0±0.3	<0.0001*
Body mass index (kg/m^2^)	24.0±0.1	24.1±0.1	0.6629	24.1±0.1		23.9±0.1	0.0451*
Waist circumference (cm)	82.8±0.1	83.7±0.4	0.0288*	82.86±0.14		82.8±0.2	0.8749
Smoking–Current smoker (%)	20.6 (0.5)	19.2 (1.8)	0.471	20.9 (0.6)		19.4 (0.1)	0.2313
Drinking–Heavy drinker (%)	9.2 (0.4)	7.1 (1.2)	0.1405	9.4 (0.4)		8.1 (0.7)	0.1365
Routine exercise (%)	19.1 (0.6)	16.7 (1.8)	0.2106	19.4 (0.6)		17.7 (0.9)	0.1253
Spouse (%)	83.4 (0.5)	79.2 (1.7)	0.0095*	84.4 (0.5)		79 (1.0)	<0.0001*
Job (%)	64.7 (0.7)	57.4 (2.1)	0.0004*	66.2 (0.7)		57.9 (1.2)	<0.0001*
Residence area–Urban (%)	76.1 (1.9)	68.4 (3.5)	0.0023*	76.1 (1.9)		73.8 (2.3)	0.0809
Education– ≥High school (%)	54.2 (0.9)	38.6 (2.2)	<0.0001*	56 (0.9)		44.2 (1.3)	<0.0001*
Income–Lower quartile (%)	21.0 (0.7)	30.6 (2.1)	<0.0001*	19.2 (0.7)		29.5 (1.2)	<0.0001*
Stress–Moderate to severe (%)	23.8 (0.5)	29.6 (2.0)	0.0030*	22.7 (0.5)		29.3 (0.9)	<0.0001*
Depressive mood (%)	14.0 (0.4)	19.6 (1.9)	0.0011*	12.8 (0.4)		19.7 (0.9)	<0.0001*
Suicidal ideation (%)	15.1 (0.4)	21.5 (1.7)	<0.0001*	13.7 (0.5)		21.2 (0.9)	<0.0001*

The data are presented as the mean±standard error or the proportion (standard error). Continuous variables were tested with the t test using the SURVEYREG and categorical variables were tested with the Rao-Scott chi-square test using the SURVEYFREQ procedure in SAS to reflct the study weights.

*Significant at *p* < 0.05

### Prevalence of olfactory dysfunction according to tinnitus and analyzed factors

**Fig 1:**
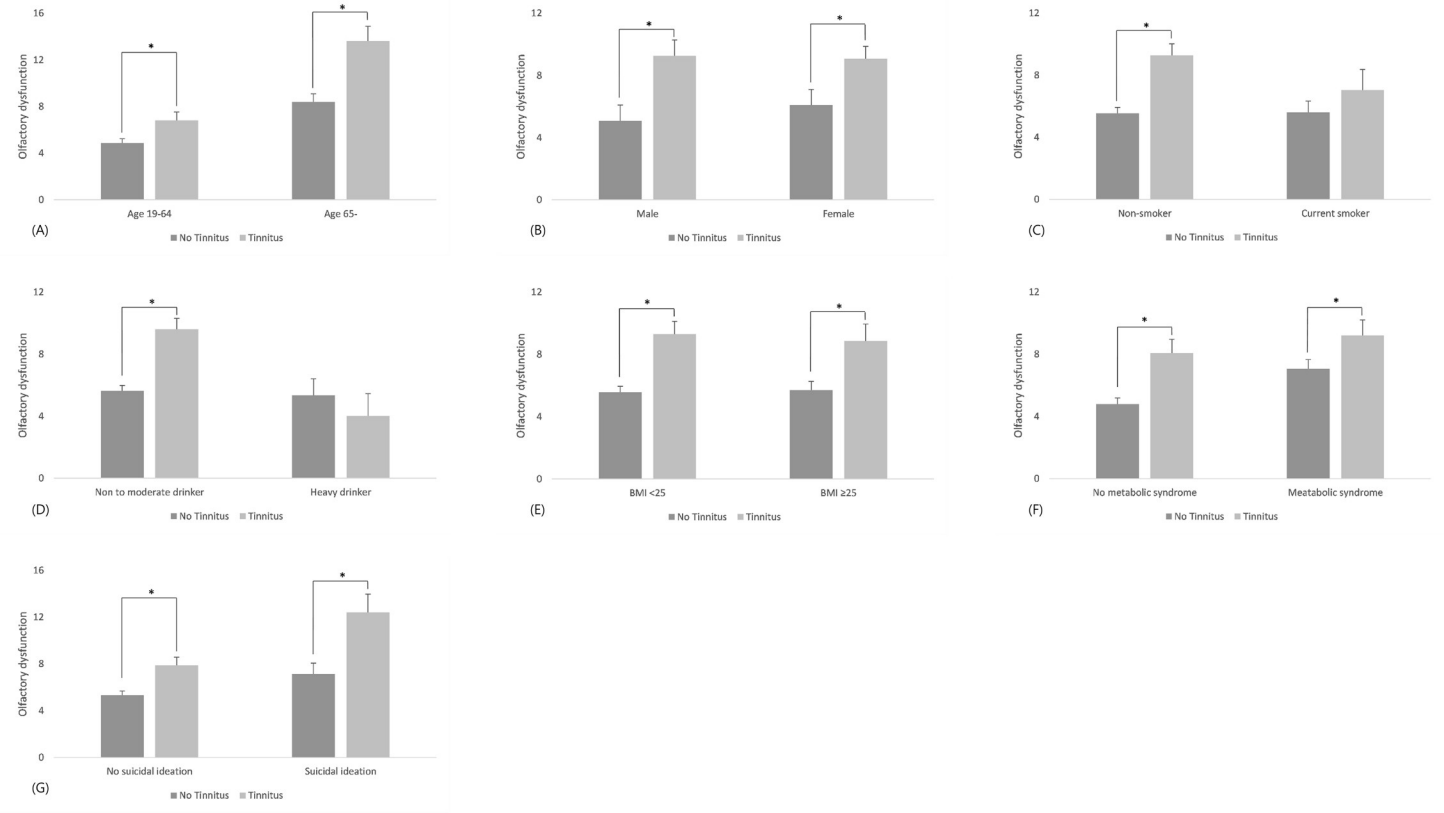
Prevalence of olfactory dysfunction according to tinnitus and analyzed factors. (A) age, (B) gender, (C) smoking history, (D) alcohol ingestion, (E) obesity, (F) metabolic syndrome, (G) suicidal ideation.

The difference in olfactory dysfunction prevalence varied statistically with age, gender, BMI, metabolic syndrome, and suicidal ideation regardless of the presence of tinnitus. Non-smokers and non- or moderate drinkers among subjects with tinnitus showed a high prevalence of olfactory dysfunction, and were distinguished from the other group. Current smokers and heavy drinkers showed no association with tinnitus or olfactory dysfunction. (Table 3, Fig 1)

**Table 3:** Prevalence of olfactory dysfunction according to tinnitus and analyzed factors.

		**Tinnitus**			**Standard errors**	
		No	Yes	*p*-value		
Age	40~64	4.8625	6.8071	0.0048*	0.3728	0.7149
	≥65	8.4078	13.6205	< .0001*	0.6742	1.2549
Gender	Male	5.0954	9.254	< .0001*	0.4642	1.0261
	Female	6.1003	9.0681	0.0002*	0.4534	0.7987
Smoking	Non-smoker	5.554	9.283	< .0001*	0.3796	0.7377
	Current smoker	5.6182	7.0555	0.2957	0.7171	1.3217
Drinking	Non to moderate drinker	5.6396	9.6035	< .0001*	0.3511	0.7079
	Heavy drinker	5.348	4.023	0.4658	1.0648	1.4319
BMI	Non-obesity (<25)	5.5694	9.3048	< .0001*	0.3813	0.8137
	Obesity (≥25)	5.7148	8.8662	0.003*	0.5561	1.0816
Metabolic syndrome	No	4.797	8.0765	< .0001*	0.3829	0.8806
	Yes	7.0628	9.2106	0.0352*	0.5939	0.9934
Suicidal ideation	No	5.321	7.8749	0.0001*	0.3614	0.7048
	Yes	7.1289	12.3995	0.0021*	0.9463	1.5807

*Significant at *p* < 0.05

### Association of olfactory dysfunction and tinnitus with metabolic syndrome and mental health

To analyze the causal relationships between the two diseases and mental health issues, the datasets of metabolic syndrome and mental health problem were analyzed with olfactory dysfunction and tinnitus. Metabolic syndrome, stress level, depressive mood, and suicidal ideation were associated with olfactory dysfunction and tinnitus (Table 4).

**Table 4:** Prevalence of metabolic syndrome and mental health problems according to the presence of olfactory dysfunction and presence and severity of tinnitus.

	**Tinnitus**					**Olfactory dysfunction**		
	No	Yes			*p*-value	No	Yes	*p*-value
		mild	moderate	severe				
Metabolic synd. (%)	35.8 (0.6)	37.0 (1.5)	44.9 (2.2)	47.0 (5.9)	<0.0001*	36.3 (0.6)	44.5 (2.1)	0.0001*
Stress level (%)	22.7 (0.5)	26.4 (1.3)	33.2 (1.7)	42.2 (5.6)	<0.0001*	23.8 (0.5)	29.6 (2.0)	0.003*
Depressive mood (%)	12.8 (0.4)	17.6 (1.1)	22.2 (1.7)	31.4 (5.0)	<0.0001*	14.0 (0.4)	19.6 (1.9)	0.0011*
Suicidal ideation (%)	13.7 (0.5)	19.1 (1.1)	23.4 (1.7)	37.3 (5.4)	<0.0001*	15.1 (0.4)	21.5 (1.7)	<0.0001*

The data are shown as the proportion (standard error).

*Significant at *p* < 0.05

### Association between olfactory dysfunction and tinnitus

Based on these results, we analyzed the relationship between olfactory dysfunction and tinnitus, and calculated the odds ratios to identify the relationship between the two ailments using multiple logistic regression models. In model 1, we adjusted for age and gender, and the odds ratio of tinnitus for olfactory dysfunction was 1.492. Increased severity of tinnitus resulted in significant increase in the odds ratio (1.251, 1.843, and 2.216). In model 2, the adjusted factors of smoking status, alcohol intake, regular exercise, income level, and education level were added to model 1, yielding an odds ratio of 1.433 for tinnitus and olfactory dysfunction. Increasing the severity of tinnitus increased the odds ratio (1.217, 1.762, and 1.874). Finally, in model 3, the adjusted factors of BMI, metabolic syndrome, DM, HTN, and stress level were added to model 2, yielding an odds ratio of 1.318 for tinnitus and olfactory dysfunction. Severe tinnitus increased the odds ratio significantly (1.134, 1.569, and 2.044) (Table 5).

**Table 5:** Logistic regression models for the association between tinnitus and olfactory dysfunction.

**Parameter**	**Model 1**			**Model 2**			**Model 3**		
	OR	95% CI	*p*-value	OR	95% CI	*p*-value	OR	95% CI	*p*-value
Tinnitus			<0.0001*			<0.0001*			0.0039*
** **No	1(ref.)			1(ref.)			1(ref.)		
** **Yes	1.492	1.252–1.779		1.433	1.199–1.712		1.318	1.093–1.590	
Tinnitus level			<0.0001*			<0.0001*			0.0004*
** **None	1(ref.)			1(ref.)			1(ref.)		
** **Mild	1.251	0.994–1.574		1.217	0.966–1.535		1.134	0.896–1.435	
** **Moderate	1.843	1.423–2.387		1.762	1.342–2.314		1.569	1.174–2.096	
** **Severe	2.216	1.277–3.843		1.874	1.013–3.467		2.044	1.070–3.906	

Model 1: Adjusted for age and gender

Model 2: Adjusted for age, gender, smoking status, alcohol intake, regular exercise, income level, and education level.

Model 3: Adjusted for age, gender, smoking status, alcohol intake, regular exercise, income level, education level, BMI, metabolic syndrome, DM, HTN, and stress level.

OR: odds ratio, CI: confidence interval

*Significant at *p*<0.05

## Discussion

The main finding of our study is that olfactory dysfunction was related to tinnitus even after adjustment for multiple factors, metabolic syndrome and comorbid psychiatric disease. Furthermore, increased severity of tinnitus was closely related to olfactory dysfunction in a dose-response relationship. Thus, our results statistically support the relationship between olfactory dysfunction and tinnitus. Previously, we reported a relationship between metabolic syndrome and olfactory dysfunction.[28] Developmentally, we analyzed the relationship between olfactory dysfunction and tinnitus, and the association with metabolic syndrome and mental health.

These results, however, must be considered taking some limitations into consideration. Hospital-based clinical olfactory tests, such as Cross-Cultural Smell Identification Test (CC-SIT), Connecticut Chemosensory Clinical Research Center (CCCRC) olfactory test, Korean Version of Sniffin' Sticks Test (KVSS), were not conducted nationwide. Tests for tinnitus were also conducted using a questionnaire-based survey, and the fact that anosmic/hyposmic or normosmic status, as well as mental health status, were also based on subjective perception of participants, and not on objective measures. So, these conditions may be the limitations of this study. Moreover, there is no consideration for the history of otorhinolaryngologic disease, such as sinus infections, middle ear dysfunction, or hearing loss. These variables may affect as additional factors accounting for variance within the relationship.

The olfactory dysfunction and tinnitus are known to be affected by a number of emotional factors in the mechanism of the disease.[8, 19, 29, 30] In this study, both tinnitus and olfactory dysfunction were associated with mental health problems. These mental health problems are likely to have played a role in the relationship between the two diseases. In addition, anti-inflammatory drugs are an important therapeutic modality when there is reasonable underlying disease in the treatment of these two diseases. Therefore, association with metabolic syndrome, which is the basic mechanism of general inflammation, also indicates the possibility of the relationship between these two diseases.[22, 23, 30] Notably, the prevalence of olfactory dysfunction in current smokers and heavy drinkers was not statistically associated with tinnitus. Probably, smoking and drinking may largely affect olfaction and tinnitus, via inflammation and other mechanisms masking the relationship between olfactory dysfunction and tinnitus.

To the best of our knowledge, there has been no study of the association between olfactory dysfunction and tinnitus based on analysis of a representative nationwide, large population-based dataset, to date. To analyze the relationship of olfactory dysfunction and tinnitus, we created three models of logistic regression adjusted for potential confounding factors, such as demographics, lifestyle habits, socioeconomic status, mental health status, and comorbid diseases.

Advances in medical science have resulted in several specialized clinical subspecialties. However, in some cases involving symptoms associated with multiple organs or syndromes, there is an increased probability of missed diagnosis based on a single subspecialty, warranting the need for multiple subspecialties and experts for a multidisciplinary approach to disease management. Paradoxically, in the world of science, the convergence of subdivided science is thought to play an important role. Therefore, the understanding of the relationship between diseases is important for diagnosis and treatment, and not merely the risk associated with individual disease.

Several studies reported the prevalence, risk factors for each disease, and the relationship with mental health.[20–23, 25, 30] To the knowledge of the authors, this is the first study to analyze statistically the association between olfactory dysfunction and tinnitus in elderly individuals. Putative pathophysiological mechanisms include infection via common passage and inflammation, although no laboratory or prospective studies are available. Therefore, further research is needed to address this question.

## Conclusions

We found the relationship between olfactory dysfunction and tinnitus. The occurrence of olfactory dysfunction may increase according to the increase in the severity of tinnitus. Additional studies are needed to unravel the mechanism and pathophysiology of the relationship between the two diseases.
